# Of mice and men: Pinpointing species differences in adipose tissue biology

**DOI:** 10.3389/fcell.2022.1003118

**Published:** 2022-09-15

**Authors:** Emma Börgeson, Jeremie Boucher, Carolina E. Hagberg

**Affiliations:** ^1^ Department of Molecular and Clinical Medicine, Wallenberg Laboratory, Institute of Medicine, Sahlgrenska Academy, University of Gothenburg, Gothenburg, Sweden; ^2^ Wallenberg Centre for Molecular and Translational Medicine, University of Gothenburg, Gothenburg, Sweden; ^3^ Region Vaestra Goetaland, Department of Clinical Physiology, Sahlgrenska University Hospital, Gothenburg, Sweden; ^4^ The Lundberg Laboratory for Diabetes Research, Department of Molecular and Clinical Medicine, Sahlgrenska Academy, University of Gothenburg, Gothenburg, Sweden; ^5^ Metabolic Disease, Evotec International GmbH, Göttingen, Germany; ^6^ Division of Cardiovascular Medicine, Department of Medicine Solna, Karolinska Institutet, Stockholm, Sweden; ^7^ Center for Molecular Medicine, Karolinska Institutet, Stockholm, Sweden

**Keywords:** obesity, adipose tissue, species differences, hypertrophy, metabolism

## Abstract

The prevalence of obesity and metabolic diseases continues to rise, which has led to an increased interest in studying adipose tissue to elucidate underlying disease mechanisms. The use of genetic mouse models has been critical for understanding the role of specific genes for adipose tissue function and the tissue’s impact on other organs. However, mouse adipose tissue displays key differences to human fat, which has led, in some cases, to the emergence of some confounding concepts in the adipose field. Such differences include the depot-specific characteristics of visceral and subcutaneous fat, and divergences in thermogenic fat phenotype between the species. Adipose tissue characteristics may therefore not always be directly compared between species, which is important to consider when setting up new studies or interpreting results. This mini review outlines our current knowledge about the cell biological differences between human and mouse adipocytes and fat depots, highlighting some examples where inadequate knowledge of species-specific differences can lead to confounding results, and presenting plausible anatomic explanations that may underlie the differences. The article thus provides critical insights and guidance for researchers working primarily with only human or mouse fat tissue, and may contribute to new ideas or concepts in the important and evolving field of adipose biology.

## Introduction

The mammalian white adipose tissue is essential for whole body lipid metabolism by storing excess meal-derived lipids as triglycerides and mobilizing them as fatty acids between meals. However, upon obesity, these functions are undermined, leading to lipids accumulating in the circulation and in peripheral organs instead, and thereby causing insulin resistance and ultimately metabolic disease (Morigny et al., 2021). The study of obesity and its detrimental consequences requires multiorgan model systems, where this complex inter-organ crosstalk can be studied and tested. The most commonly used model is rodents, and especially mice. Genetically modified mice have for example been key for improving our understanding of how obesity affects adipose tissue cellularity (Wang et al., 2013), how changes in fat cell (adipocyte) number versus size impact systemic metabolism (Kim et al., 2007; Wang et al., 2008), and for discovering genes that affect body weight and/or systemic insulin resistance, with the best-known example being the discovery of the *obese* or leptin gene (Zhang et al., 1994). However, when the ultimate goal is to translate these findings to human pathophysiology, a clearer understanding of species differences, and the potential limitations of these murine model systems, is needed. The fact remains that many aspects of adipocyte biology differ significantly between mice and humans, which is not always acknowledged. This mini review aims to outline some of the most important aspects of how human and mouse visceral and subcutaneous adipose depots differ, highlighting how inadequate knowledge of species differences easily can lead to confounding concepts, and how to avoid such confusion. We hope it can serve as a guide for researchers new to the adipose field who mostly work solely with either human or mouse material. We also think that acknowledging species differences could bring new ideas about adipose biology and help us identify which adipocyte characteristics drive the development of adipose tissue dysfunction during obesity, and which adipocyte characteristics merely correlating with it.

## Divergent characteristics of mouse and human adipose depots

### The basis of all confusion—Different fat pad organization

In general, the white adipose tissue (WAT) depots can, for both mice and humans, be divided into two major anatomical regions, subcutaneous and visceral fat. Subcutaneous fat is found just beneath the skin, and visceral fat is located within the central body cavity. Whereas obesity is associated with an overall negative impact on health and increased mortality, the expansion of subcutaneous fat in humans has been shown to be associated with beneficial or neutral effects on metabolism, whereas excess visceral fat correlates with both metabolic and cardiovascular risk factors (Goodpaster et al., 1997; Lotta et al., 2017). The subcutaneous fat is therefore considered the physiological site for lipid storage, that for lean humans typically comprises the majority, around 80%, of the total body fat (Sakers et al., 2022).

**FIGURE 1:**
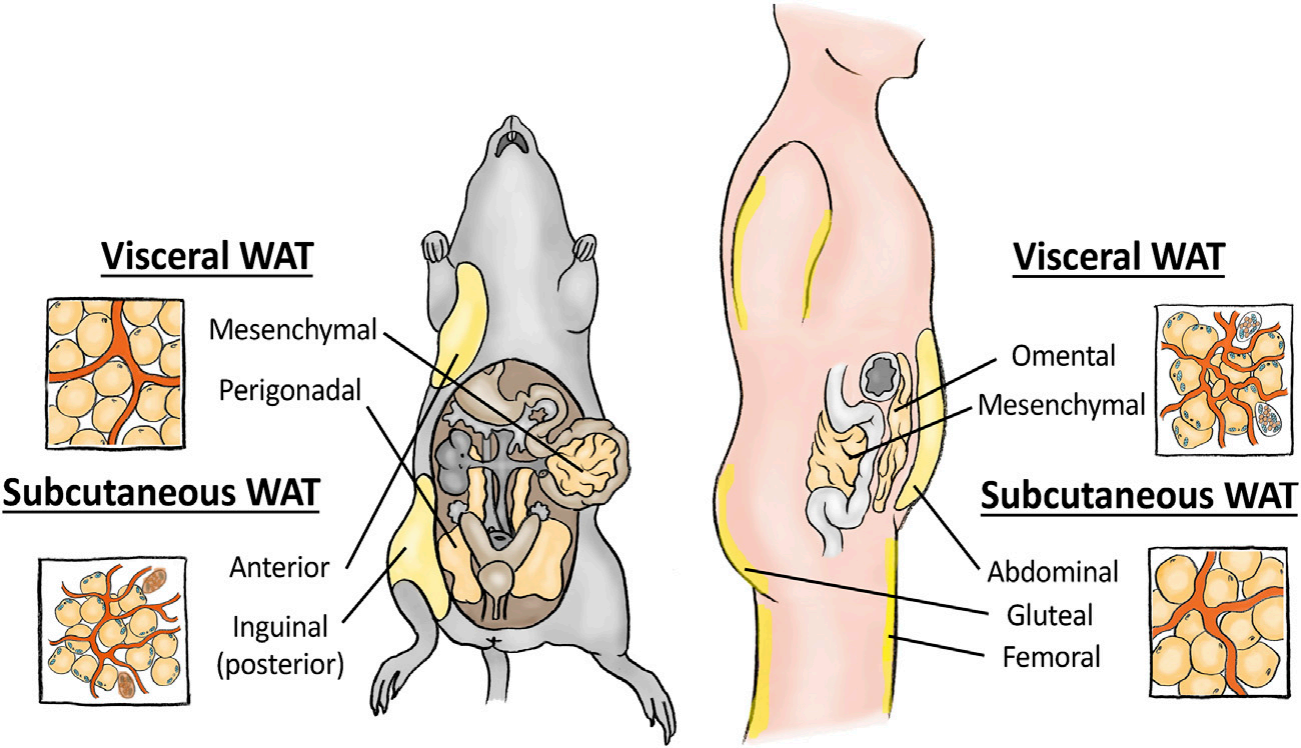
Schematic showing the most important visceral and subcutaneous white adipose tissue depots in mice (left) and humans (right), respectively. Enlarged simplified drawings of the respective tissue structures are merely indicative of the relative differences between depots, and do not include all details of the tissue itself.

These two major fat depots can be further subdivided into specific fat pads, and here confusion may arise. In mice, subcutaneous fat is mainly found in the posterior inguinal WAT (iWAT) depot and in the anterior axillary region (Figure 1). Humans similarly develop subcutaneous fat in the abdominal region (abWAT) but also in the femoral (thigh) and gluteal (bum) region, the latter especially in women (Chusyd et al., 2016). Human abdominal subcutaneous fat can further be divided into a superficial and a deep subcutaneous layer, whereas mice most likely lack this anatomical division (Chusyd et al., 2016). Despite these slight differences, most researchers agree that the majority of results from mouse iWAT and human abWAT are largely comparable. It is important to note that the subcutaneous fat is the major physiological storage location for meal-derived lipids, and only when its expansion capacity is exceeded do lipids start to accumulate in visceral fat. This is clearly illustrated in patients with almost any kind of lipodystrophy, where genetically induced loss of subcutaneous fat, especially of that in the gluteal and femoral depots, recapitulates most features of the metabolic syndrome, whereas their amount of visceral fat is not correlated to disease severity (Mann and Savage, 2019).

For visceral fat pads, the direct comparison between species is less correct. In mice, the most well studied visceral fat depot is the peri-gonadal white adipose tissue (gWAT, is also sometimes termed epididymal fat in males, and periovarian fat in females), which dominates their central cavity (Figure 1). Mice also have peri-renal, some mesenteric and some omental visceral WAT, but the latter two mouse fat pads are of negligible size and their impact on systemic metabolism could be questioned (Vitali et al., 2012). The gWAT has therefore remained the best studied fat pad and is in rodent studies often referred to just as visceral fat. Importantly, humans almost completely lack this fat pad, and instead organize most of their visceral fat into the omental WAT (oWAT), with mesenteric and peri-renal fat being the other two dominating human visceral fat pads (Chusyd et al., 2016). Accumulation of human oWAT has in numerous studies been shown to associate with metabolic disease and increased risk for developing comorbidities such as insulin resistance, dyslipidaemia, type-2 diabetes and cardiovascular disease (Zhang et al., 2015; Chait and den Hartigh, 2020). In mice, the expansion of visceral gWAT fat is similarly associated with metabolic dysfunction and whole-body insulin resistance (Gabriely et al., 2002), and these correlations could have given rise to the misguiding notion that these two visceral fat pads are comparable. However, these fat pads exhibit numerous important differences. The human oWAT is a large flat adipose tissue layer hanging down as a “curtain” or an elastic apron from the stomach to the liver, floating on top of the small and large intestine and other visceral organs (Di Nicola, 2019). Despite its tight correlation with metabolic disease, its main physiological role in addition to lipid storage is to control anatomical infection and isolate wounds (Di Nicola, 2019). The tissue in lean, healthy patients therefore contains high levels of immune cells, including macrophages, B- and T-cells, often found concentrated to milky spots within the tissue. The second important human visceral fat region is the mesenteric fat, that wraps around the intestine (Zwick et al., 2018). In both humans and mice, the mesenteric fat is thought to function similarly to the omentum, storing lipids and upholding the intestinal barrier. However, this fat is small in mice, and difficult to extract in humans due to its high degree of vascularization, and thus remains understudied, as does peri-renal white adipose tissue for both species (Wernstedt Asterholm et al., 2014; Chusyd et al., 2016). Instead, when studying visceral fat, mouse gWAT is often considered to correspond to human oWAT due to their abdominal locations. However, gWAT is located adjacent to the mouse sex organs and as such maintains none of the immunomodulatory functions of human omental and mesenteric fat, but is rather thought to function mainly as a cushion for the mouse reproductive organs in addition to storing lipids (Chusyd et al., 2016). Moreover, while the human visceral oWAT drains directly to the portal circulation, mouse gWAT drains to the systemic circulation, which greatly impacts the influence that respective fat depot has on the liver (Rytka et al., 2011). In addition, lipid tracing experiments in humans have shown that while human oWAT directly drains to the liver, lipids released from this fat pad constitute only a small proportion of the total lipid released by WAT, suggesting that oWAT-derived pro-inflammatory adipokines rather than lipid release may mediate the negative effects of having excess visceral fat (Jensen, 2008; Rytka et al., 2011). For more detailed information on the specific anatomical fat pad organizations and their differences between mice and men we refer to two recent excellent reviews (Chusyd et al., 2016; Di Nicola, 2019), whereas the focus below will be on the more cell biological differences that exists between these two major fat pads in mice and humans.

### Human and mouse fat pads show opposite patterns of adipocyte size

During weight gain, WAT expands both as a result of resident adipocytes growing in size, leading to a more *hypertrophic* tissue, but also via the emergence of new fat cells through the differentiation of tissue-resident progenitor cells termed pre-adipocytes, resulting in a more *hyperplastic* adipose tissue. Whereas adipocyte hypertrophy has been suggested to dominate the initial phase of human weight gain (Salans et al., 1971; Spalding et al., 2008), elegant pulse-chase studies showed new adipocytes being formed within the mouse visceral gWAT fat after approximately 8 weeks of high fat diet feeding (Wang et al., 2013). Mouse subcutaneous iWAT seemed to expand only via hypertrophy, analogous to the human tissue. Importantly, these two modes of tissue expansion, hypertrophy and hyperplasia, associate very differently with insulin resistance and other metabolic risk factors. Adipocyte hypertrophy and increased adipocyte size have by numerous studies been identified as one of the best markers of a dysfunctional WAT, associated with an increased risk for development of insulin resistance, dyslipidemia and metabolic disease even in non-obese individuals (Acosta et al., 2016; Verboven et al., 2018; Rawshani et al., 2020). Transgenic mice that develop hypertrophic obesity, such as the *ob/ob* and *db/db* mice, typically develop metabolic dysfunctions, whereas severely obese mice with hyperplastic fat containing many small adipocytes instead are able to remain relatively healthy (Shepherd et al., 1993; Boucher et al., 2002; Kim et al., 2007; Kim et al., 2014). Some notable exceptions to this exist such as when collagen 6 is knocked out, which causes severely hypertrophic obesity without apparent metabolic defects, potentially because the reduced levels of restraining collagen in the adipocytes’ microenvironment allow them to take up and store more lipid (Khan et al., 2009). In addition to large hypertrophic adipocytes most often being an adverse marker of metabolic health, mouse visceral adipocytes are also larger than subcutaneous cells (Johnson and Hirsch, 1972), contributing to the wrongful conception that *absolute* adipocyte size is a determinant of adipocyte dysfunction. It is therefore important to recognize that in humans the adipocyte size relationship between fat pads is *the opposite*, with the more beneficial abdominal subcutaneous fat displaying larger fat cell sizes, especially in lean individuals, than the human visceral oWAT (Belligoli et al., 2019; O'Connell et al., 2010). Moreover, human femoral subcutaneous adipocytes, which are considered to possess the most beneficial impact on systemic metabolism, are even larger than abWAT from the same subject, further contradicting the notion of a large absolute size being detrimental for adipocyte functionality (Tchoukalova et al., 2008; Vogel et al., 2019). Interestingly, several studies comparing adipocyte size in humans with a wide range of body weights show that whereas human subcutaneous abWAT display larger cell sizes than that of visceral oWAT in healthy lean subjects, the relative increase in size/volume of visceral oWAT cells is larger during weight gain (Verboven et al., 2018; O'Connell et al., 2010; Suarez-Cuenca et al., 2021). In rodents, this pattern seems to be the same (Gabriely et al., 2002). Thus, the *relative growth of adipocytes*, as compared to cells from the same anatomical location of controls, may represent a better marker of metabolic dysfunction than absolute adipocyte size *per se*. However, taking the above differences in account, most generalizations from comparing subcutaneous to visceral adipocyte size between species should on the whole be avoided.

### Adipocyte characteristics primarily follow adipocyte size, not anatomical location

The species-specific pattern of adipocyte sizes has far more important consequences for adipocyte biology than one realizes at first glance. Several studies have confirmed (directly or indirectly) that the larger subcutaneous adipocyte size in humans is associated with a higher degree of adipocyte insulin resistance (Virtanen et al., 2002; Lundgren et al., 2004; Laviola et al., 2006) and lower mitochondrial content (Kraunsoe et al., 2010) in human abWAT as compared to oWAT. Exceptions do exist and the results may depend on the degree of obesity or measurement method. Human abWAT also takes up glucose less efficiently than oWAT, as evaluated by PET imaging or uptake of radioactive glucose (Stolic et al., 2002; Lundgren et al., 2004; Christen et al., 2010). This is the opposite to what several studies have shown for mouse fat pads (Wueest et al., 2012; Schottl et al., 2015a; Schottl et al., 2015b). Again, notable exceptions do exist, and it is unclear what underlies these discrepancies (Macotela et al., 2009). These species differences imply that adipocyte size might be a more important determinant of some of these characteristics than anatomical location. In addition, some of these adipocyte characteristics may not have adverse effects on adipocyte function in humans, as expansion of the more (insulin resistant) human subcutaneous fat in general is not considered to have adverse effects on systemic metabolic health (Lotta et al., 2017). One of the underlying mechanisms for the altered subcutaneous insulin sensitivity could be that as the adipocytes accumulate increased amounts of triglycerides in their lipid droplets, their plasma membrane becomes somewhat stretched, potentially influencing local concentrations of signalling receptors and thereby influencing insulin receptor dimerization upon ligand binding (Livingston et al., 1984). This may of course be context dependent and thus remains highly debated. It should also be noted that some studies suggest that the various signalling pathways downstream of the insulin receptor could be differentially affected by obesity, leading to some pathways being more efficiently blocked by insulin resistance compared to others (Farnier et al., 2003). Thus, whether adipocyte insulin sensitivity is assessed by measuring insulin-stimulated glucose uptake, or as insulin-induced receptor phosphorylation may lead to different results (Lundgren et al., 2004; Laviola et al., 2006).

Importantly, in adipocytes, insulin signalling does not only regulate glucose uptake, but also functions as the main inhibitor of fatty acid release from adipocytes by limiting intracellular lipolysis. Therefore, adipocyte insulin resistance can also be measured as sensitivity to insulin-mediated inhibition of lipolysis. Again, reports suggest that human visceral oWAT adipocytes are more insulin sensitive than subcutaneous adipocytes in this regard as well, and that subcutaneous adipocytes therefore have a higher basal lipolytic activity compared to oWAT cells from matched individuals (Arner, 1995; Hoffstedt et al., 1997; van Harmelen et al., 2002). Another important example of depot and species differences is the differential regulation of catecholamine-induced adipocyte lipolysis (Tchernof et al., 2006; Lafontan and Langin, 2009). Different adipose tissue depots express varying levels of the pro-lipolytic beta1, beta2 and beta3 adrenergic receptors, as well as the antilipolytic alpha2A adrenergic receptor, and their relative balance is determinant for each depot’s lipolytic activity. Human adipocytes from the subcutaneous (especially gluteal and femoral) depots have a higher expression of antilipolytic alpha2A adrenergic receptors compared to human visceral depots, and thus display lower catecholamine-induced lipolysis (Mauriege et al., 1987; Castan et al., 1993; Lafontan and Berlan, 1995). Rodent adipocytes on the other hand express very few alpha2A adrenergic receptors in any of their depots (Castan et al., 1994), and their catecholamine-induced lipolysis is mainly mediated via activation of the pro-lipolytic beta3 adrenergic receptor (Lafontan, 1994). In human adipocytes the beta3 adrenergic receptor is expressed at very low levels, and instead catecholamine-induced lipolytic activation is mediated via the beta1 and beta2 adrenergic receptors (Tavernier et al., 1996). Taken together, these results highlight key differences between human and mouse adipose tissue depot characteristics with regards to insulin sensitivity and control of lipolysis.

Another important characteristic that is influenced by adipocyte size is vascular density when measured per unit volume. Increased adipocyte size is inversely correlated to vascular density, as larger cell sizes lead to resident capillaries being pushed further apart and the inter-capillary distance thereby increasing. Obesity-induced adipocyte enlargement therefore leads to a lower capillary density in all human and mouse fat pads, which subsequently can cause hypoxia and WAT fibrosis (Belligoli et al., 2019; Lempesis et al., 2020). However, due to the intrinsic differences in adipocyte size discussed above, there is already a difference in the basal capillary density between fat pads. Again, due to human abWAT adipocytes being larger than visceral oWAT adipocytes, the human subcutaneous fat has a lower vascular density (Ledoux et al., 2008; Villaret et al., 2010; Belligoli et al., 2019), with the relationship between the mouse depots being the opposite (Song et al., 2016). Decreased oxygen tension, a direct measurement of hypoxia, has been suggested to tightly follow vascular density in mice, whereas the occurrence of WAT hypoxia in human fat has been harder to measure, especially for oWAT (Cifarelli et al., 2020; Lempesis et al., 2020). In summary, by comparing measurements from human fat pads, displaying the opposite pattern of adipocyte sizes than in mice, we could potentially be able to differentiate between adipose tissue characteristics that directly promote metabolic disease, and those that merely correlate with it.

### Different thermogenic pattern between the two species

In addition to WAT, rodents also possess brown adipose tissue (BAT), which, as the name alludes to, is more brown-looking and is a thermogenic fat type that specializes in dissipating the energy from lipids and other nutrients as heat instead of solely storing them. BAT is found primarily in the interscapular region in mice, and gets its colour from its high mitochondrial content and vascularization. In addition to bona fide BAT can mouse subcutaneous fat, and to a much lesser extent visceral gWAT, upon chronic stimulation of cold or adrenergic agonists acquire brown-like features, with increased lipid oxidation and thermogenic capacity (Vitali et al., 2012; Herz and Kiefer, 2019). These thermogenic cells can arise both from resident progenitor cells and via transdifferentiation of white adipocytes, and are referred to as brite/beige adipocytes due to their intrinsic differences to bona fide brown adipocytes (Maurer et al., 2021). In more recent years have human adults also been shown to possess thermogenic adipocytes, found predominantly concentrated around larger vessels such as in the supraclavicular, paravertebral, periaortic and axillary regions (Cypess et al., 2009; van Marken Lichtenbelt et al., 2009; Virtanen et al., 2009). In fact, human supraclavicular fat possesses an equally high thermogenic capacity as mouse BAT when expressed per mitochondrion (Porter et al., 2016). However, when measuring the expression of thermogenic and mitochondrial genes within the major human WAT depots, the anatomic expression pattern of these genes is again the opposite from that of mice, with human visceral oWAT and mediastinal fat showing higher thermogenic expression than human abWAT (Cheung et al., 2013; Zuriaga et al., 2017). Although not explicitly shown, this is most likely due to different amounts of beige/brown cells between depots, and not the levels of expression per cell. Whether the browning capacity also is different between the depots remains to be established in humans. Lastly, another significant difference between humans and mice is that human brown adipocytes do not readily express the major murine adrenergic receptor, the beta3 adrenergic receptor, and instead rely on stimulation via only the beta2 adrenergic receptor, and therefore the two species have different responses to such stimulations (Blondin et al., 2020). Taken together, the brown adipose tissue field has recently published a number of interesting papers detailing the species differences between mice and humans, and using this information to better translate knowledge gained from animal models to future clinical applications in humans (Kowaltowski, 2022).

## Concluding remarks and potential future developments in the field

### How a deeper knowledge of species-specific differences can avoid confounding results and help advance obesity research

Taken together, it is clear that mice are not men, but also that many of our insights in adipose tissue biology come from rodent studies. How can we best utilize these differences to our advantage and avoid confusion? First of all, we should be aware of these differences, and also make others aware by always including the detailed anatomical location of any studied fat pad in articles and abstracts, using for example oWAT and gWAT throughout the text instead of simply visceral WAT. The recent publication of single cell sequencing data of several fat depots from both mice and humans will greatly contribute to the comparison of adipose biology between the species and promises to become an important resource for translational studies (Emont et al., 2022). Secondly, we should more readily start to accept and even embrace adipose tissue species differences, as they can teach us about different aspects of adipose biology and its relationship to metabolic diseases. This includes more readily publishing and discussing results that differ between species, for the field to learn what part of adipose biology is directly translatable between species, and what is not. This may also involve reviewers being more careful when asking for validation of mouse data in human material and vice versa, accepting that not all results can be directly compared, but still can contribute to valuable knowledge. Thirdly, considering the species differences, we should more readily try to use human material in our studies, instead of relying on mouse *in vitro* and *in vivo* models. Tools to study human adipocytes have increased dramatically in the past decade, with more human primary or immortalized adipocyte cells becoming available, more commercial vendors of such cells, more fresh adipose biopsies being available, and the development of more sophisticated methods to grow, differentiate and maintain human adipocytes (Dufau et al., 2021). This includes Membrane Mature Adipocyte Aggregate Cultures (MAAC) for long term culturing of mature adipocytes (Harms et al., 2019), and 3D-culturing models for differentiating adipocyte progenitors *in vitro* using either scaffold-free conditions (Shen et al., 2021), or a scaffold that allows adipocytes to differentiate along vascular sprouts and form human unilocular vascularized adipose spheroids (HUVAS) (Ioannidou et al., 2022). While rodent models remain a vital and unexchangeable part of adipose tissue research, these advances have now opened the door for more labs to do translational research and continue exploring the differences and similarities in metabolism between species.

## References

[B1] Acosta J. R. Douagi I. Andersson D. P. Backdahl J. Ryden M. Arner P. (2016). Increased fat cell size: A major phenotype of subcutaneous white adipose tissue in non-obese individuals with type 2 diabetes. Diabetologia 59 (3), 560–570. 10.1007/s00125-015-3810-6 26607638

[B2] Arner P. (1995). Differences in lipolysis between human subcutaneous and omental adipose tissues. Ann. Med. 27 (4), 435–438. 10.3109/07853899709002451 8519504

[B3] Belligoli A. Compagnin C. Sanna M. Favaretto F. Fabris R. Busetto L. (2019). Characterization of subcutaneous and omental adipose tissue in patients with obesity and with different degrees of glucose impairment. Sci. Rep. 9 (1), 11333. 10.1038/s41598-019-47719-y 31383894 PMC6683173

[B4] Blondin D. P. Nielsen S. Kuipers E. N. Severinsen M. C. Jensen V. H. Miard S. (2020). Human Brown adipocyte thermogenesis is driven by β2-AR stimulation. Cell Metab. 32 (2), 287–300. 10.1016/j.cmet.2020.07.005 32755608

[B5] Boucher J. Castan-Laurell I. Le Lay S. Grujic D. Sibrac D. Krief S. (2002). Human alpha 2A-adrenergic receptor gene expressed in transgenic mouse adipose tissue under the control of its regulatory elements. J. Mol. Endocrinol. 29 (2), 251–264. 10.1677/jme.0.0290251 12370125

[B6] Castan I. Valet P. Larrouy D. Voisin T. RemAury A. DaviauD D. (1993). Distribution of PYY receptors in human fat cells: An antilipolytic system alongside the alpha 2-adrenergic system. Am. J. Physiol. 265 (1), E74–E80. 10.1152/ajpendo.1993.265.1.E74 8393293

[B7] Castan I. Valet P. Quideau N. Voisin T. Ambid L. LaburtheM. (1994). Antilipolytic effects of alpha 2-adrenergic agonists, neuropeptide Y, adenosine, and PGE1 in mammal adipocytes. Am. J. Physiol. 266 (4), R1141–R1147. 10.1152/ajpregu.1994.266.4.R1141 7910434

[B8] Chait A. den Hartigh L. J. (2020). Adipose tissue distribution, inflammation and its metabolic consequences, including diabetes and cardiovascular disease. Front. Cardiovasc. Med. 7, 22. 10.3389/fcvm.2020.00022 32158768 PMC7052117

[B9] Cheung L. Gertow J. Werngren O. FoLkersen L. PetrovicN. Nedergaard J. (2013). Human mediastinal adipose tissue displays certain characteristics of brown fat. Nutr. Diabetes 3, e66. 10.1038/nutd.2013.6 23670224 PMC3671748

[B10] Christen T. Sheikine Y. Rocha V. Z. Hurwitz S. Goldfine A. B. Di Carli M. (2010). Increased glucose uptake in visceral versus subcutaneous adipose tissue revealed by PET imaging. JACC. Cardiovasc. Imaging 3 (8), 843–851. 10.1016/j.jcmg.2010.06.004 20705265 PMC4042675

[B11] Chusyd D. E. Wang D. Huffman D. M. Nagy T. R. (2016). Relationships between rodent white adipose fat pads and human white adipose fat depots. Front. Nutr. 3, 10. 10.3389/fnut.2016.00010 27148535 PMC4835715

[B12] Cifarelli V. Beeman S. C. Smith G. I. Yoshino J. Morozov D. Beals J. W. (2020). Decreased adipose tissue oxygenation associates with insulin resistance in individuals with obesity. J. Clin. Invest. 130 (12), 6688–6699. 10.1172/JCI141828 33164985 PMC7685757

[B13] Cypess A. M. Lehman S. Williams G. Tal I. Rodman D. Goldfine A. B. (2009). Identification and importance of Brown adipose tissue in adult humans. N. Engl. J. Med. 360 (15), 1509–1517. 10.1056/NEJMoa0810780 19357406 PMC2859951

[B14] Di Nicola V. (2019). Omentum a powerful biological source in regenerative surgery. Regen. Ther. 11, 182–191. 10.1016/j.reth.2019.07.008 31453273 PMC6700267

[B15] Dufau J. Shen J. X. Couchet M. De Castro Barbosa T. Mejhert N. Massier L. (2021). *In vitro* and *ex vivo* models of adipocytes. Am. J. Physiol. Cell Physiol. 320 (5), C822–C841. 10.1152/ajpcell.00519.2020 33439778

[B16] Emont M. P. Jacobs C. Essene A. L. Pant D. Tenen D. Colleluori G. (2022). A single-cell atlas of human and mouse white adipose tissue. Nature 603 (7903), 926–933. 10.1038/s41586-022-04518-2 35296864 PMC9504827

[B17] Farnier C. Krief S. Blache M. Diot-DupuyF. Mory G. Ferre P. (2003). Adipocyte functions are modulated by cell size change: Potential involvement of an integrin/ERK signalling pathway. Int. J. Obes. Relat. Metab. Disord. 27 (10), 1178–1186. 10.1038/sj.ijo.0802399 14513065

[B18] Gabriely I. Ma X. H. Yang X. M. Atzmon G. Rajala M. W. Berg A. H. (2002). Removal of visceral fat prevents insulin resistance and glucose intolerance of aging: An adipokine-mediated process? Diabetes 51 (10), 2951–2958. 10.2337/diabetes.51.10.2951 12351432

[B19] Goodpaster B. H. Thaete F. L. Simoneau J. A. Kelley D. E. (1997). Subcutaneous abdominal fat and thigh muscle composition predict insulin sensitivity independently of visceral fat. Diabetes 46 (10), 1579–1585. 10.2337/diacare.46.10.1579 9313753

[B20] Harms M. J. Li Q. Lee S. Zhang C. Kull B. Hallen S. (2019). Mature human white adipocytes cultured under membranes maintain identity, function, and can transdifferentiate into Brown-like adipocytes. Cell Rep. 27 (1), 213–225. 10.1016/j.celrep.2019.03.026 30943403

[B21] Herz C. T. Kiefer F. W. (2019). Adipose tissue browning in mice and humans. J. Endocrinol. 241 (3), R97–R109. 10.1530/joe-18-0598 31144796

[B22] Hoffstedt J. Arner P. Hellers G. Lonnqvist F. (1997). Variation in adrenergic regulation of lipolysis between omental and subcutaneous adipocytes from obese and non-obese men. J. Lipid Res. 38 (4), 795–804. 10.1016/s0022-2275(20)37246-1 9144094

[B23] Ioannidou A. Alatar S. Schipper R. Baganha F. Ahlander M. Hornell A. (2022). Hypertrophied human adipocyte spheroids as *in vitro* model of weight gain and adipose tissue dysfunction. J. Physiol. 600 (4), 869–883. 10.1113/JP281445 34387376

[B24] Jensen M. D. (2008). Role of body fat distribution and the metabolic complications of obesity. J. Clin. Endocrinol. Metab. 93 (11), S57–S63. 10.1210/jc.2008-1585 18987271 PMC2585758

[B25] Johnson P. R. Hirsch J. (1972). Cellularity of adipose depots in six strains of genetically obese mice. J. Lipid Res. 13 (1), 2–11. 10.1016/s0022-2275(20)39428-1 5059196

[B26] Khan T. Muise E. S. Iyengar P. Wang Z. V. Chandalia M. Abate N. (2009). Metabolic dysregulation and adipose tissue fibrosis: Role of collagen VI. Mol. Cell. Biol. 29 (6), 1575–1591. 10.1128/MCB.01300-08 19114551 PMC2648231

[B27] Kim J. Y. van de Wall E. Laplante M. Azzara A. Trujillo M. E. Hofmann S. M. (2007). Obesity-associated improvements in metabolic profile through expansion of adipose tissue. J. Clin. Invest. 117 (9), 2621–2637. 10.1172/JCI31021 17717599 PMC1950456

[B28] Kim S. M. Lun M. Wang M. Senyo S. E. Guillermier C. Patwari P. (2014). Loss of white adipose hyperplastic potential is associated with enhanced susceptibility to insulin resistance. Cell Metab. 20 (6), 1049–1058. 10.1016/j.cmet.2014.10.010 25456741 PMC4715375

[B29] Kowaltowski A. J. (2022). Cold exposure and the metabolism of mice, men, and other wonderful creatures. Physiol. (Bethesda) 37 (5), 0. 10.1152/physiol.00002.2022 35575253

[B30] Kraunsoe R. Boushel R. Hansen C. N. Schjerling P. Qvortrup K. Stockel M. (2010). Mitochondrial respiration in subcutaneous and visceral adipose tissue from patients with morbid obesity. J. Physiol. 588 (12), 2023–2032. 10.1113/jphysiol.2009.184754 20421291 PMC2911209

[B31] Lafontan M. Berlan M. (1995). Fat cell alpha 2-adrenoceptors: The regulation of fat cell function and lipolysis. Endocr. Rev. 16 (6), 716–738. 10.1210/edrv-16-6-716 8747832

[B32] Lafontan M. (1994). Differential recruitment and differential regulation by physiological amines of fat cell beta-1, beta-2 and beta-3 adrenergic receptors expressed in native fat cells and in transfected cell lines. Cell. Signal. 6 (4), 363–392. 10.1016/0898-6568(94)90085-x 7946963

[B33] Lafontan M. Langin D. (2009). Lipolysis and lipid mobilization in human adipose tissue. Prog. Lipid Res. 48 (5), 275–297. 10.1016/j.plipres.2009.05.001 19464318

[B34] Laviola L. Perrini S. Cignarelli A. Natalicchio A. Leonardini A. De Stefano F. (2006). Insulin signaling in human visceral and subcutaneous adipose tissue *in vivo* . Diabetes 55 (4), 952–961. 10.2337/diabetes.55.04.06.db05-1414 16567516

[B35] Ledoux S. Queguiner I. Msika S. Calderari S. Rufat P. Gasc J. M. (2008). Angiogenesis associated with visceral and subcutaneous adipose tissue in severe human obesity. Diabetes 57 (12), 3247–3257. 10.2337/db07-1812 18835936 PMC2584130

[B36] Lempesis I. G. van Meijel R. L. J. Manolopoulos K. N. Goossens G. H. (2020). Oxygenation of adipose tissue: A human perspective. Acta Physiol. 228 (1), e13298. 10.1111/apha.13298 PMC6916558 31077538

[B37] Livingston J. N. Lerea K. M. Bolinder J. Kager L. Backman L. Arner P. (1984). Binding and molecular weight properties of the insulin receptor from omental and subcutaneous adipocytes in human obesity. Diabetologia 27 (4), 447–453. 10.1007/BF00273909 6391988

[B38] Lotta L. A. Gulati P. Day F. R. Payne F. Ongen H. van de Bunt M. (2017). Integrative genomic analysis implicates limited peripheral adipose storage capacity in the pathogenesis of human insulin resistance. Nat. Genet. 49 (1), 17–26. 10.1038/ng.3714 27841877 PMC5774584

[B39] Lundgren M. Buren J. Ruge T. Myrnas T. Eriksson J. W. (2004). Glucocorticoids down-regulate glucose uptake capacity and insulin-signaling proteins in omental but not subcutaneous human adipocytes. J. Clin. Endocrinol. Metab. 89 (6), 2989–2997. 10.1210/jc.2003-031157 15181089

[B40] Macotela Y. Boucher J. Tran T. T. Kahn C. R. (2009). Sex and depot differences in adipocyte insulin sensitivity and glucose metabolism. Diabetes 58 (4), 803–812. 10.2337/db08-1054 19136652 PMC2661589

[B41] Mann J. P. Savage D. B. (2019). What lipodystrophies teach us about the metabolic syndrome. J. Clin. Invest. 129 (10), 4009–4021. 10.1172/JCI129190 31380809 PMC6763226

[B42] Maurer S. Harms M. Boucher J. (2021). The colorful versatility of adipocytes: white-to-brown transdifferentiation and its therapeutic potential in humans. FEBS J. 288 (12), 3628–3646. 10.1111/febs.15470 32621398

[B43] Mauriege P. Galitzky J. Berlan M. Lafontan M. (1987). Heterogeneous distribution of beta and alpha-2 adrenoceptor binding sites in human fat cells from various fat deposits: Functional consequences. Eur. J. Clin. Invest. 17 (2), 156–165. 10.1111/j.1365-2362.1987.tb02395.x 3034620

[B44] Morigny P. Boucher J. Arner P. Langin D. (2021). Lipid and glucose metabolism in white adipocytes: Pathways, dysfunction and therapeutics. Nat. Rev. Endocrinol. 17 (5), 276–295. 10.1038/s41574-021-00471-8 33627836

[B45] O'Connell J. Lynch L. Cawood T. J. Kwasnik A. Nolan N. Geoghegan J. (2010). The relationship of omental and subcutaneous adipocyte size to metabolic disease in severe obesity. PLoS One 5 (4), e9997. 10.1371/journal.pone.0009997 20376319 PMC2848665

[B46] Porter C. Herndon D. N. Chondronikola M. Chao T. Annamalai P. Bhattarai N. (2016). Human and mouse Brown adipose tissue mitochondria have comparable UCP1 function. Cell Metab. 24 (2), 246–255. 10.1016/j.cmet.2016.07.004 27508873 PMC5201422

[B47] Rawshani A. Eliasson B. Rawshani A. Henninger J. Mardinoglu A. Carlsson A. (2020). Adipose tissue morphology, imaging and metabolomics predicting cardiometabolic risk and family history of type 2 diabetes in non-obese men. Sci. Rep. 10 (1), 9973. 10.1038/s41598-020-66199-z 32561768 PMC7305301

[B48] Rytka J. M. Wueest S. Schoenle E. J. Konrad D. (2011). The portal theory supported by venous drainage-selective fat transplantation. Diabetes 60 (1), 56–63. 10.2337/db10-0697 20956499 PMC3012197

[B49] Sakers A. De Siqueira M. K. Seale P. Villanueva C. J. (2022). Adipose-tissue plasticity in health and disease. Cell 185 (3), 419–446. 10.1016/j.cell.2021.12.016 35120662 PMC11152570

[B50] Salans L. B. Horton E. S. Sims E. A. (1971). Experimental obesity in man: Cellular character of the adipose tissue. J. Clin. Invest. 50 (5), 1005–1011. 10.1172/JCI106570 5552403 PMC292021

[B51] Schottl T. Kappler L. Braun K. Fromme T. Klingenspor M. (2015). Limited mitochondrial capacity of visceral versus subcutaneous white adipocytes in male C57BL/6N mice. Endocrinology 156 (3), 923–933. 10.1210/en.2014-1689 25549046

[B52] Schottl T. Kappler L. Fromme T. Klingenspor M. (2015). Limited OXPHOS capacity in white adipocytes is a hallmark of obesity in laboratory mice irrespective of the glucose tolerance status. Mol. Metab. 4 (9), 631–642. 10.1016/j.molmet.2015.07.001 26413469 PMC4563017

[B53] Shen J. X. Couchet M. Dufau J. de Castro Barbosa T. Ulbrich M. H. Helmstadter M. (2021). 3D adipose tissue culture links the organotypic microenvironment to improved adipogenesis. Adv. Sci. 8 (16), e2100106. 10.1002/advs.202100106 PMC8373086 34165908

[B54] Shepherd P. R. Gnudi L. Tozzo E. Yang H. LeachF. Kahn B. B. (1993). Adipose cell hyperplasia and enhanced glucose disposal in transgenic mice overexpressing GLUT4 selectively in adipose tissue. J. Biol. Chem. 268 (30), 22243–22246. 10.1016/s0021-9258(18)41516-5 8226728

[B55] Song M. G. Lee H. J. Jin B. Y. Gutierrez-Aguilar R. Shin K. H. Choi S. H. (2016). Depot-specific differences in angiogenic capacity of adipose tissue in differential susceptibility to diet-induced obesity. Mol. Metab. 5 (11), 1113–1120. 10.1016/j.molmet.2016.09.001 27818937 PMC5081408

[B56] Spalding K. L. Arner E. Westermark P. O. Bernard S. Buchholz B. A. Bergmann O. (2008). Dynamics of fat cell turnover in humans. Nature 453 (7196), 783–787. 10.1038/nature06902 18454136

[B57] Stolic M. Russell A. Hutley L. Hay J. MacDonald G. Whitehead J. (2002). Glucose uptake and insulin action in human adipose tissue--influence of BMI, anatomical depot and body fat distribution. Int. J. Obes. Relat. Metab. Disord. 26 (1), 17–23. 10.1038/sj.ijo.0801850 11791142

[B58] Suarez-Cuenca J. A. De La Pena-Sosa G. De La Vega-Moreno K. Banderas-Lares D. Z. Salamanca-Garcia M. Martinez-Hernandez J. E. (2021). Enlarged adipocytes from subcutaneous vs. visceral adipose tissue differentially contribute to metabolic dysfunction and atherogenic risk of patients with obesity. Sci. Rep. 11 (1), 1831. 10.1038/s41598-021-81289-2 33469087 PMC7815822

[B59] Tavernier G. Barbe P. Galitzky J. BerlanM. Caput D. LafontanM. (1996). Expression of beta3-adrenoceptors with low lipolytic action in human subcutaneous white adipocytes. J. Lipid Res. 37 (1), 87–97. 10.1016/s0022-2275(20)37638-0 8820105

[B60] Tchernof A. Belanger C. Morisset A. S. Richard C. Mailloux J. Laberge P. (2006). Regional differences in adipose tissue metabolism in women: Minor effect of obesity and body fat distribution. Diabetes 55 (5), 1353–1360. 10.2337/db05-1439 16644692

[B61] Tchoukalova Y. D. Koutsari C. Karpyak M. V. Votruba S. B. Wendland E. Jensen M. D. (2008). Subcutaneous adipocyte size and body fat distribution. Am. J. Clin. Nutr. 87 (1), 56–63. 10.1093/ajcn/87.1.56 18175737

[B62] van Harmelen V. Dicker A. Ryden M. Hauner H. Lonnqvist F. Naslund E. (2002). Increased lipolysis and decreased leptin production by human omental as compared with subcutaneous preadipocytes. Diabetes 51 (7), 2029–2036. 10.2337/diabetes.51.7.2029 12086930

[B63] van Marken Lichtenbelt W. D. Vanhommerig J. W. Smulders N. M. Drossaerts J. M. A. F. L. Kemerink G. J. Bouvy N. D. (2009). Cold-activated Brown adipose tissue in healthy men. N. Engl. J. Med. 360 (15), 1500–1508. 10.1056/NEJMoa0808718 19357405

[B64] Verboven K. Wouters K. Gaens K. Hansen D. BijnenM. WetzelS S. (2018). Abdominal subcutaneous and visceral adipocyte size, lipolysis and inflammation relate to insulin resistance in male obese humans. Sci. Rep. 8 (1), 4677. 10.1038/s41598-018-22962-x 29549282 PMC5856747

[B65] Villaret A. Galitzky J. Decaunes P. Esteve D. Marques M. A. Sengenes C. (2010). Adipose tissue endothelial cells from obese human subjects: Differences among depots in angiogenic, metabolic, and inflammatory gene expression and cellular senescence. Diabetes 59 (11), 2755–2763. 10.2337/db10-0398 20713685 PMC2963533

[B66] Virtanen K. A. Lidell M. E. Orava J. Heglind M. Westergren R. Niemi T. (2009). Functional Brown adipose tissue in healthy adults. N. Engl. J. Med. 360 (15), 1518–1525. 10.1056/NEJMoa0808949 19357407

[B67] Virtanen K. A. Lonnroth P. Parkkola R. Peltoniemi P. Asola M. Viljanen T. (2002). Glucose uptake and perfusion in subcutaneous and visceral adipose tissue during insulin stimulation in nonobese and obese humans. J. Clin. Endocrinol. Metab. 87 (8), 3902–3910. 10.1210/jcem.87.8.8761 12161530

[B68] Vitali A. Murano I. Zingaretti M. C. Frontini A. Cinti S. (2012). The adipose organ of obesity-prone C57BL/6J mice is composed of mixed white and Brown adipocytes. J. Lipid Res. 53 (4), 619–629. 10.1194/jlr.M018846 22271685 PMC3307639

[B69] Vogel M. A. A. Wang P. Bouwman F. G. HoebersN. Blaak E. E. Renes J. (2019). A comparison between the abdominal and femoral adipose tissue proteome of overweight and obese women. Sci. Rep. 9 (1), 4202. 10.1038/s41598-019-40992-x 30862933 PMC6414508

[B70] Wang M. Y. Grayburn P. Chen S. Ravazzola M. Orci L. Unger R. H. (2008). Adipogenic capacity and the susceptibility to type 2 diabetes and metabolic syndrome. Proc. Natl. Acad. Sci. U. S. A. 105 (16), 6139–6144. 10.1073/pnas.0801981105 18413598 PMC2329698

[B71] Wang Q. A. Tao C. Gupta R. K. Scherer P. E. (2013). Tracking adipogenesis during white adipose tissue development, expansion and regeneration. Nat. Med. 19 (10), 1338–1344. 10.1038/nm.3324 23995282 PMC4075943

[B72] Wernstedt Asterholm I. Tao C. Morley T. S. Wang Q. A. Delgado-Lopez F. Wang Z. V. (2014). Adipocyte inflammation is essential for healthy adipose tissue expansion and remodeling. Cell Metab. 20 (1), 103–118. 10.1016/j.cmet.2014.05.005 24930973 PMC4079756

[B73] Wueest S. Schoenle E. J. Konrad D. (2012). Depot-specific differences in adipocyte insulin sensitivity in mice are diet- and function-dependent. Adipocyte 1 (3), 153–156. 10.4161/adip.19910 23700524 PMC3609098

[B74] Zhang M. Hu T. Zhang S. Zhou L. (2015). Associations of different adipose tissue depots with insulin resistance: A systematic review and meta-analysis of observational studies. Sci. Rep. 5, 18495. 10.1038/srep18495 26686961 PMC4685195

[B75] Zhang Y. Proenca R. Maffei M. BaroneM. LeopoLd L. Friedman J. M. (1994). Positional cloning of the mouse obese gene and its human homologue. Nature 372 (6505), 425–432. 10.1038/372425a0 7984236

[B76] Zuriaga M. A. Fuster J. J. Gokce N. Walsh K. (2017). Humans and mice display opposing patterns of "browning" gene expression in visceral and subcutaneous white adipose tissue depots. Front. Cardiovasc. Med. 4, 27. 10.3389/fcvm.2017.00027 28529941 PMC5418233

[B77] Zwick R. K. Guerrero-Juarez C. F. Horsley V. Plikus M. V. (2018). Anatomical, physiological, and functional diversity of adipose tissue. Cell Metab. 27 (1), 68–83. 10.1016/j.cmet.2017.12.002 29320711 PMC6050204

